# A new species of the spider genus *Khorata* Huber, 2005 (Araneae, Pholcidae), with a list of *Khorata* species from Vietnam

**DOI:** 10.3897/BDJ.12.e128884

**Published:** 2024-07-09

**Authors:** Ludan Zhang, Shuqiang Li, Zhiyuan Yao

**Affiliations:** 1 College of Life Science, Shenyang Normal University, Shenyang, China College of Life Science, Shenyang Normal University Shenyang China; 2 Institute of Zoology, Chinese Academy of Sciences, Beijing, China Institute of Zoology, Chinese Academy of Sciences Beijing China

**Keywords:** biodiversity, checklist, daddy-long-legs, morphology, taxonomy

## Abstract

**Background:**

The genus *Khorata* Huber, 2005 contains 51 species. It is distributed in the Indo-Malayan Region. Nine species have been recorded from Vietnam.

**New information:**

*Khorataninhbinh* sp. nov. is described as a new species from Vietnam. In addition, a list of all *Khorata* species from Vietnam is also provided.

## Introduction

The genus *Khorata* Huber, 2005, belonging to the subfamily Pholcinae C.L. Koch, 1850, was first established by Huber (2005). Currently, 51 species of the genus *Khorata* have been recorded (World Spider Catalog 2024). Of them, 34 are from southern China (e.g. Tong and Li (2008), Zhang and Zhang (2008), Zhang and Zhu (2009), Yao and Li (2010), Yao et al. (2019), Xu et al. (2020)), nine are from Vietnam (Yao et al. 2015, Nie et al. 2018), six are from Laos (Huber 2005, Yao and Li 2013), four are from Thailand (Huber 2005, Yao et al. 2014, Lan et al. 2021) and one is from Cambodia (Lan et al. 2021). The records from Kep, Cambodia represent the southernmost distribution limit for the genus (Lan et al. 2021).

The aim of this work is to describe a new species of *Khorata* and provide a list of this genus from Vietnam (Table 1).

## Materials and methods

Specimens were examined and measured with a Leica M205 C stereomicroscope. The left male palp was photographed. The epigyne was photographed before dissection. The vulva was photographed after treating it in a 10% warm solution of potassium hydroxide (KOH) to dissolve soft tissues. Images were captured with a Canon EOS 750D wide zoom digital camera (24.2 megapixels) mounted on the stereomicroscope mentioned above and assembled using Helicon Focus v. 3.10.3 image stacking software (Khmelik et al. 2005). All measurements are given in millimetres (mm). Leg measurements are shown as: total length (femur, patella, tibia, metatarsus and tarsus). Leg segments were measured on their dorsal sides. The specimens studied are preserved in 75% ethanol and deposited in the College of Life Science, Shenyang Normal University (SYNU) in Liaoning, China.

Terminology and taxonomic descriptions follow Huber (2005), Yao et al. (2021) and Lu et al. (2022). The following abbreviations are used in the descriptions: **ALE** = anterior lateral eye, **AME** = anterior median eye, **PME** = posterior median eye, **L/d** = length/diameter; used in the illustrations: **aa** = anterior arch, **b** = bulb, **da** = distal apophysis, **e** = embolus, **fa** = frontal apophysis, **pa** = proximo-lateral apophysis, **pp** = pore plate, **pr** = procursus.

## Taxon treatments

### Khorata ninhbinh sp. nov.

BD0CF44F-3ACC-5228-BACD-54B31191FC41

BD47BBD6-1C03-499A-97FD-631378B465A0

#### Materials

- **Type status:** Holotype. **Occurrence:** recordedBy: S Li, G Zheng, DS Pham; individualCount: 1; sex: male; lifeStage: adult; occurrenceID: 45700102-0BF5-599E-83A5-782455060ED4; **Taxon:** order: Araneae; family: Pholcidae; genus: Khorata; **Location:** country: Vietnam; stateProvince: Ninh Binh; locality: Cuc Phuong National Park; verbatimLocality: Mat Cave; verbatimElevation: 18 m a.s.l.; verbatimLatitude: 20°21.125’N; verbatimLongitude: 105°12.381’E; **Event:** year: 2008; month: 7; day: 22; **Record Level:** institutionCode: SYNU-Ar00411
- **Type status:** Paratype. **Occurrence:** recordedBy: S Li, G Zheng, DS Pham; individualCount: 3; sex: 1 male, 2 females; lifeStage: adult; occurrenceID: C61182A2-DC30-5006-900F-E1CC69BFFBF8; **Taxon:** order: Araneae; family: Pholcidae; genus: Khorata; **Location:** country: Vietnam; stateProvince: Ninh Binh; locality: Cuc Phuong National Park; verbatimLocality: Mat Cave; verbatimElevation: 18 m a.s.l.; verbatimLatitude: 20°21.125’N; verbatimLongitude: 105°12.381’E; **Event:** year: 2008; month: 7; day: 22; **Record Level:** institutionCode: SYNU-Ar00412–00414

#### Description

**Figure 1.**
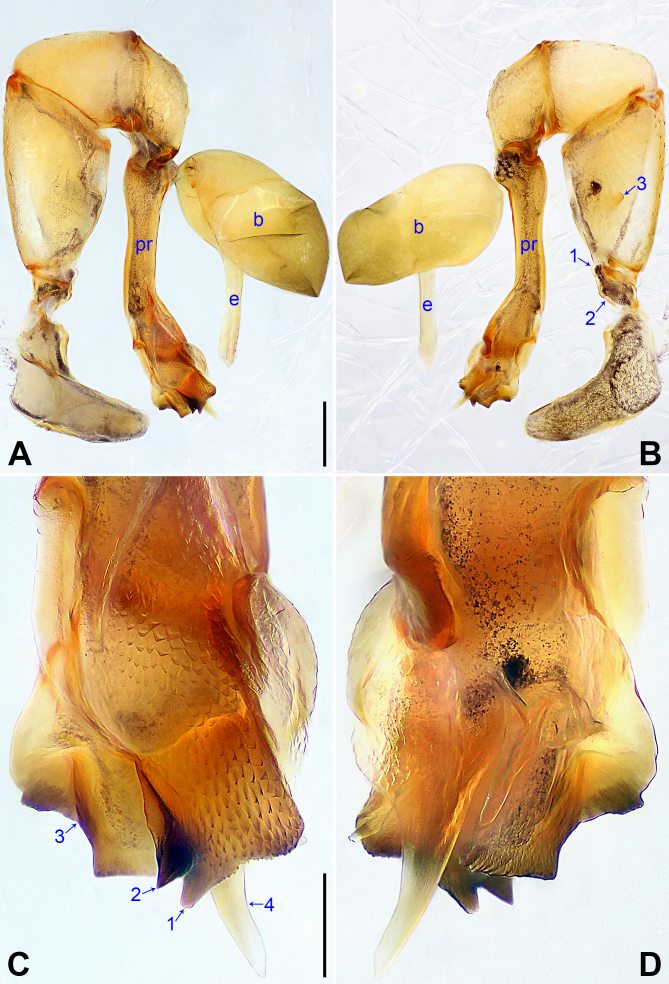
*Khorataninhbinh* sp. nov., holotype male **A, B** palp (**A** prolateral view, **B** retrolateral view, arrow 1 points at trochanteral retrolateral apophysis, arrow 2 points at trochanteral ventral apophysis, arrow 3 points at femoral retrolateral apophysis); **C, D** distal part of procursus (**C** prolateral view, arrows 1, 2 point at angular apophyses, arrow 3 points at retrolateral sclerite, arrow 4 points at retrolatero-distal apophysis, **D** retrolateral view). Abbreviations: b = bulb, e = embolus, pr = procursus. Scale bars: 0.20 (**A, B**); 0.05 (**C, D**).

**Figure 2.**
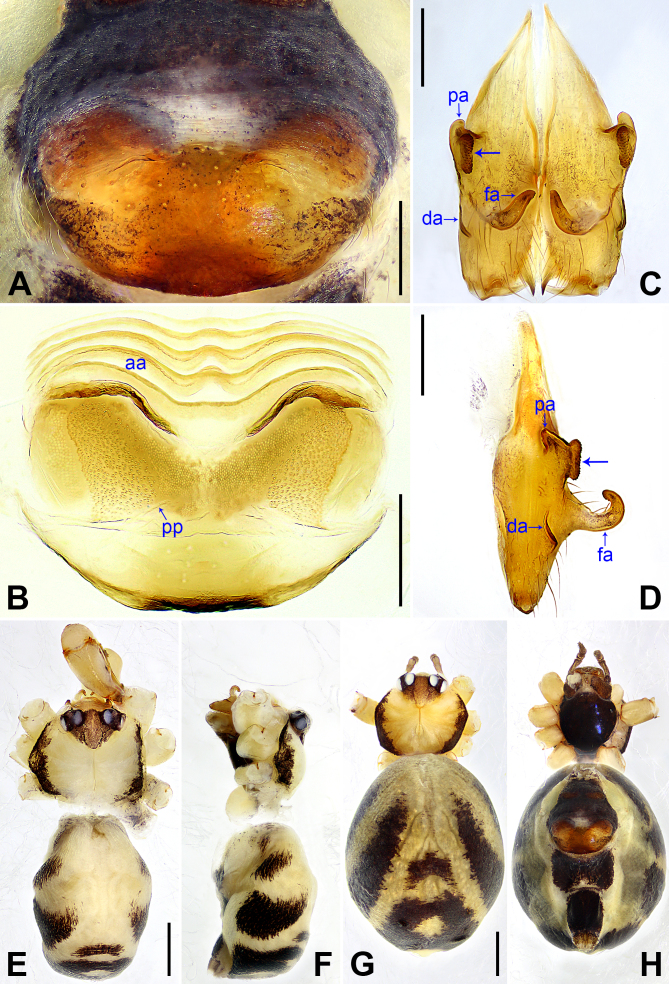
*Khorataninhbinh* sp. nov., holotype male (**C–F**) and paratype female (**A, B, G, H**) **A** epigyne, ventral view; **B** vulva, dorsal view; **C, D** chelicerae (**C** frontal view, **D** lateral view; arrows point at strong frontal apophyses); **E–H** habitus (**E, G** dorsal view, **F** lateral view, **H** ventral view). Abbreviations: aa = anterior arch, da = distal apophysis, fa = frontal apophysis, pa = proximo-lateral apophysis, pp = pore plate. Scale bars: 0.20 (**A–D**); 0.50 (**E–H**).

**Male** (***holotype***): Total length 2.62 (2.71 with clypeus), prosoma 0.85 long, 1.06 wide, opisthosoma 1.77 long, 1.12 wide. Leg I: 31.83 (7.91, 0.43, 7.88, 13.01, 2.60), legs II and III missing, leg IV: 19.55 (5.77, 0.42, 4.75, 7.37, 1.24); tibia I L/d: 66. Eye interdistances and diameters: PME–PME 0.16, PME 0.14, PME–ALE 0.04, AME absent. Sternum width/length: 0.78/0.66. Habitus as in Fig. 2E and F. Dorsal shield of prosoma yellowish, with black lateral margins and narrow, dark median line; sternum black. Legs brownish, but slightly whitish on distal parts of femora and tibiae, with distinct darker rings on subdistal parts of femora and tibiae. Opisthosoma yellowish, with large black spots. Thoracic furrow shallow, but distinct. Clypeus unmodified. Ocular area slightly elevated and separated from rest of prosoma. Chelicerae with pair of proximo-lateral apophyses (pa in Fig. 2C and D), pair of distal apophyses (da in Fig. 2C and D) on front-lateral surface, pair of strong frontal apophyses (arrows in Fig. 2C and D) each bearing scales and pair of long, hooked frontal apophyses (fa in Fig. 2C and D; distance between tips: 0.05). Palp as in Fig. 1A and B; trochanter with short retrolateral apophysis (as long as wide, arrow 1 in Fig. 1B) and small ventral apophysis (arrow 2 in Fig. 1B); femur with small retrolateral apophysis (arrow 3 in Fig. 1B); patella large; procursus simple proximally, but complex distally, with prolateral distal apophysis bearing scales and two angular apophyses (arrows 1 and 2 in Fig. 1C), curved retrolateral sclerite (arrow 3 in Fig. 1C) and spine-shaped, weakly sclerotised retrolatero-distal apophysis (arrow 4 in Fig. 1C); bulb simple, no other apophyses, except for embolus. Retrolateral trichobothria on tibia I at 6% proximally; legs with short vertical setae on metatarsi and tarsi; tarsus I with 12 distinct pseudosegments.

**Female** (***paratype***, SYNU-Ar00413): Similar to male, habitus as in Fig. 2G and H. Total length 3.20 (3.36 with clypeus), prosoma 0.90 long, 1.05 wide, opisthosoma 2.30 long, 1.78 wide; leg I missing. Eye interdistances and diameters: PME–PME 0.17, PME 0.14, PME–ALE 0.03, AME absent. Sternum width/length: 0.64/0.62. Epigyne (Fig. 2A) sclerotised and posteriorly slightly curved, without pockets. Vulva with wavy anterior arch (aa in Fig. 2B) and pair of anteriorly blunt and posteriorly pointed pore plates (nearly elliptic, pp in Fig. 2B).

**Variation.** Tibia I in the male paratype (SYNU-Ar00412): 7.88. Tibia I in another female paratype (SYNU-Ar00414): 7.69.

#### Diagnosis

The new species resembles *K.bachma* Yao & Li, 2018 (Nie et al. 2018: 481, figs 1A–D, 2A–H) by having similar male chelicerae (Fig. 2C and D) and epigyne (Fig. 2A), but can be distinguished by procursus with two angular prolateral apophyses (arrows 1 and 2 in Fig. 1C vs. nearly half-round apophysis in *K.bachma*), curved retrolateral sclerite (arrow 3 in Fig. 1C vs. square in *K.bachma*) and spine-shaped, weakly sclerotised retrolatero-distal apophysis (arrow 4 in Fig. 1C vs. absent in *K.bachma*), by pore plates anteriorly blunt and posteriorly pointed (nearly elliptic, pp in Fig. 2B vs. triangular in *K.bachma*) and by vulval anterior arch wavy (aa in Fig. 2B vs. straight in *K.bachma*).

#### Etymology

The specific name refers to the type locality; noun in apposition.

#### Distribution

Vietnam (Ninh Binh, type locality).

#### Biology

The species was found in the twilight zone (entrance ecotone) of the Mat Cave.

## Supplementary Material

XML Treatment for Khorata ninhbinh

## Figures and Tables

**Table 1.** A list of all *Khorata* species from Vietnam.

**Species**	**Habitat**	**Reference**
*K.bachma* Yao & Li, 2018	web between rocks	Nie et al. (2018)
*K.cucphuong* Yao & Li, 2018	web between rocks	Nie et al. (2018)
*K.dangi* Yao, Pham & Li, 2015	cave entrance	Yao et al. (2015)
*K.digitata* Yao & Li, 2010	cave entrance	Yao et al. (2015)
*K.huberi* Yao, Pham & Li, 2015	cave entrance	Yao et al. (2015)
*K.ninhbinh* **sp. nov.**	cave entrance	this paper
*K.palace* Yao & Li, 2018	cave entrance	Nie et al. (2018)
*K.protumida* Yao, Pham & Li, 2015	cave entrance	Yao et al. (2015)
*K.quangbinh* Yao & Li, 2018	web between rocks	Nie et al. (2018)
*K.vinhphuc* Yao & Li, 2018	web between rocks	Nie et al. (2018)
